# Influence of seasonal variation on in vitro fertilization success

**DOI:** 10.1371/journal.pone.0199210

**Published:** 2018-07-05

**Authors:** Michal Kirshenbaum, Alon Ben-David, Eran Zilberberg, Tal Elkan-Miller, Jigal Haas, Raoul Orvieto

**Affiliations:** 1 Department of Obstetrics and Gynecology, Chaim Sheba Medical Center, Tel-Hashomer, Ramat Gan, Israel; 2 Sackler Faculty of Medicine, Tel Aviv University, Tel Aviv, Israel; 3 The Tarnesby-Tarnowski Chair for Family Planning and Fertility Regulation, Sackler Faculty of Medicine, Tel-Aviv University, Israel; University of Florida, UNITED STATES

## Abstract

**Objective:**

To evaluate the influence of seasonal variation on in vitro fertilization (IVF) outcome in a large cohort population.

**Methods & materials:**

A total of 5,765 IVF cycles conducted in Sheba medical center between 2013 and 2016 were retrospectively analyzed. The treatment cycles included 4214 ovarian stimulation and ovum pick up (OPU) cycles of which 3020 resulted in fresh embryo transfer and 1551 vitrified- warmed cycles of which1400 resulted in warmed embryo transfer. Cycles were assigned to seasons according to the date of OPU for fresh embryo transfer cycles or according to the date of embryo warming for vitrified warmed embryo transfer cycles.

**Results:**

There were no statistically significant differences between the calendar months or seasons concerning the number of oocytes retrieved or fertilization rate in the fresh cycles. Throughout the 4 years of the study, the monthly clinical pregnancy rate fluctuated between 18.2% and 27.9% per fresh embryo transfer (mean 23.3%) and between 17.7% and 29.4% per vitrified warmed embryo transfer (mean 23%). These fluctuations did not follow any specific seasonal pattern.

**Conclusions:**

Our study did not demonstrate any significant influence of the calendar months or seasons on the clinical pregnancy rates of fresh or vitrified warmed embryo transfers. It might be speculated that the complete pharmaceutical control of the ovarian and endometrial function, as well as the homogeneous treatments, procedures and laboratory equipment used during the study period have lowered the influence of seasonal effect on IVF treatment outcome.

## Introduction

Several epidemiologic studies throughout the world have demonstrated seasonal changes in natural conception and birth rate [1][2]. The seasonal related factors affecting natural conception include sperm quality, ovulation rate and social parameters. Evidence suggests that deterioration in sperm quality during the summer in sub-equatorial areas may result in lower conception rate and a reduction in birth rate in the spring [3][4]. Moreover, previous observations demonstrated an effect of environmental light exposure on female reproductive axis, ovulation and endometrial receptivity [5][6]. These observations were attributed to the variance in melatonin secretion, which increases in the darkness. In addition to the biologic seasonal variation, social parameters and patterns of sexual activity may also influence reproduction and birth rate in human [2][7].

Data regarding the seasonal variation during assisted reproductive treatment is controversial. In in vitro fertilization (IVF), fluctuation in endogenous gonadotropins secretion is irrelevant due to the GnRH-analogues induced pituitary suppression, as part of controlled ovarian hyperstimulation (COH). Nonetheless, seasonal conditions may still influence the fertilization rate or embryo quality, as was suggested by Rojansky et al (8). They demonstrated a significant seasonal variability, with the highest fertilization rate and best quality embryos during the spring and the lowest in the autumn, correlated with the absolute number of light hours [8]. Likewise, Wood et al showed a significantly improved implantation rate and greater clinical pregnancy rate during summer cycles [9]. Vanderkerckhove et al demonstrated higher live birth rates if the patients were exposed to more sunshine hours during the month before treatment [10]. These observations might be explained by the influence of melatonin on different levels of the reproductive tract. On the other hand, other studies failed to demonstrate any specific seasonal patterns regarding IVF treatment outcomes. Gindes et al [11] and Revelli et al [12] found that neither fertilization, nor implantation rate in fresh embryo transfer cycles were affected by the season of the year. To avoid bias in the fertilization rate, Wunder et al excluded cycles using ICSI and examined only IVF cycles, showing no effect of seasons on treatment outcome [13]. Fleming et al found no difference in fertilization rate between seasons; nevertheless, a trend towards higher implantation rate was seen in the autumn [14].

The aforementioned inconsistency of associations between seasonality and IVF treatment outcome may derive from differences in the inclusion criteria, treatment protocols, laboratory equipment or geographical area. However, it may also derive from different seasonality effects on the multiple factors consisting the female reproductive tract. Because the hypothalamic- pituitary function is suppressed in assisted reproductive treatment, the seasonal effect is expressed as the end organ function, which is translated to fertilization rate and endometrial receptivity. A separate investigation of fresh or vitrified warmed embryo transfer cycles might isolate the seasonal effect on endometrial receptivity and its influence on IVF outcome.

Prompted by these observations we aimed to investigate the influence of seasonal variation on IVF outcome in a tertiary, university-affiliated medical center, with stratification to fresh and vitrified warmed embryo transfer cycles.

## Materials and methods

All consecutive patients undergoing assisted reproduction cycles resulting in ovum pick up (OPU) or vitrified warmed embryo transfer cycles, between January 2013 and December 2016, were included in the study analysis. The selection COH protocol or the type of endometrial preparation used, were the decision of the treating physician and largely dependent on the fashion at the time. The protocols of ovarian stimulation follow up and oocyte retrieval have been described elsewhere [15]. In the vitrifies warmed cycles, patients underwent either natural cycle or assisted hormone replacement as described in a previous study conducted in our institution [16]. The insemination media used was "Global total for insemination" (Life Global, Guilford, CT, USA). The culture and embryo transfer media used was "Continuous Single Culture- Complex" (Irvine Scientific, Santa Ana, Ca, USA). During the study period, there were no differences in therapeutic protocols, laboratory methodology, techniques or equipment nor embryologists' skills. All embryos were cryopreserved by vitrification, using a vitrification kit (SAGE Vitrification Kit, SAGE Media, USA). Embryo classification in our institution during the study period was based on the individual embryo scoring parameters according to pre-established definitions, while a top quality embryo is defined as seven or more blastomeres on day 3, equally-sized blastomeres and <20% fragmentation, poor quality embryos consist of all the rest [17].

Ethics statement: The study was approved by the Institutional Research Ethics Board of Sheba Medical Center (SMC 4850–18). Data collection was conducted anonymously and informed consent was not required.

Data regarding the number of OPU procedures, vitrified warmed cycles, embryo transfers and pregnancy rate was prospectively collected. Outcome was assessed in terms of number of eggs retrieved per OPU cycle, rate of fertilization, positive beta-human chorionic gonadotropin test rate, clinical pregnancy rate and miscarriage rate. The clinical pregnancy rate was defined as the proportion of patients with one or more intrauterine gestational sacs demonstrated by transvaginal ultrasound 4 weeks after embryo transfer. In order to differentiate the impact of seasonal variation on endometrial receptivity from that of ovum and embryo quality, the clinical pregnancy rate was calculated separately for the fresh embryo transfer cycles and for the vitrified warmed embryo cycles. Results were compared between individual calendar months each year. The months were grouped to seasons which were defined before the analysis of data according to the calendar definition of seasons for Israel, each season lasting 3 months: spring: March-May, summer: June- August, fall: September- November, winter: December- February.

The attribution of the patients to the corresponding seasons was made according to the date of the OPU for fresh embryo transfer cycles or according to the date of embryo warming for vitrified warmed embryo cycles.

SPSS version 24 was used for statistical analysis. Monthly rate and means were calculated for the different variables. The effect of seasons and calendar months on cycle outcomes was examined using one way ANOVA for normally distributed variables and Kruskal-Wallis test for non-normally distributed variables. Two way ANOVA was conducted to examine the effect of interaction between season and cycle type (fresh or thawed) on the rate of clinical pregnancies.

## Results

**Fig 1:**
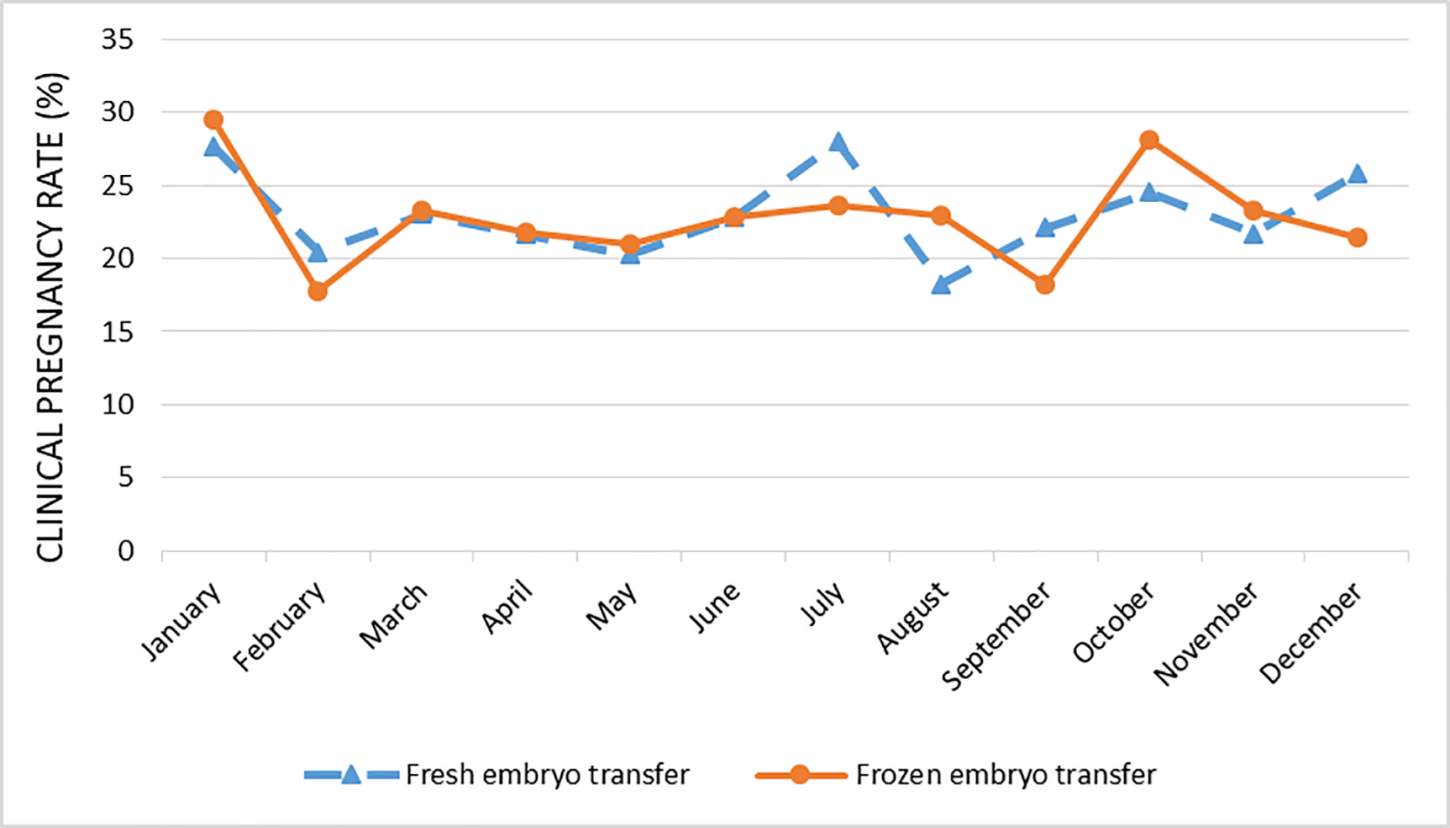
Pregnancy rate by calendar month for the four consecutive years (2013–2016).

Between January 2013 and December 2016, 5765 assisted reproduction treatment cycles were performed in the IVF unit in Sheba medical center and were included in the analysis. The treatment cycles included 4214 ovarian stimulation and ovum pick up (OPU) cycles of which 3020 resulted in fresh embryo transfer and 1551 vitrified- warmed cycles of which1400 resulted in warmed embryo transfer. The overall clinical pregnancy rate during the study period was 23.3% for fresh embryo transfer cycles and 23% for vitrified warmed cycles (p = 0.74). Table 1 and Fig 1 describe the distribution of the treatment cycles and the clinical pregnancy rates in each calendar month for the four consecutive years 2013–2016. There was no difference between the consecutive years regarding the number OPU cycles, number of fresh or frozen embryo transfers or clinical pregnancy rates.

**Table 1:** Distribution of treatment cycles and pregnancy rates per embryo transfer.

	Fresh cycles 2013–2016			Vitrified-warmed cycles 2013–2016		
	No. of OPU	No. of fresh embryo transfer	Clinical pregnancy n (%)	No. of warmings	No. of warmed embryo transfer	Clinical pregnancy n (%)
**January**	389	275	76 (27.6)	123	112	33 (29.4)
**February**	343	264	54 (20.4)	108	96	17 (17.7)
**March**	392	278	64 (23)	120	116	27 (23.2)
**April**	325	245	53 (21.6)	123	115	25 (21.7)
**May**	339	247	50 (20.4)	137	129	27 (21)
**June**	358	272	62 (22.7)	115	105	24 (22.8)
**July**	430	293	82 (27.9)	150	123	29 (23.5)
**August**	153	104	19 (18.2)	92	87	20 (23)
**September**	358	267	59 (22.1)	112	99	18 (18.2)
**October**	366	261	64 (24.5)	143	135	38 (28.1)
**November**	354	235	51 (21.70	154	129	30 (23.2)
**December**	407	279	72 (26)	174	154	33 (21.4)
**TOTAL**	4214	3020	706 (23.3)	1551	1400	321 (23)

Monthly and seasonal analysis of the fresh embryo transfer cycles did not demonstrate any significant differences regarding patients' age or COH variables. The mean number of oocytes retrieved per OPU, fertilization rate, mean number of embryos transferred and the clinical pregnancy rates per embryo transfer did not significantly differ between the seasons (Table 2).

**Table 2:** Treatment cycles of fresh embryo transfer stratified by seasons.

	TOTAL	Spring	Summer	Autumn	Winter	P-VALUE
**Mean age of women (years) mean±SD**	35.7±5.7	36.3±5.6	35.9±5.7	35.5±5.6	35.2±5.8	NS
**No. of OPU cycles mean ± SD**	263.4 ± 27.0	264.0 ± 24.1	235.2 ± 21.3	269.5 ±21.5	284.7 ± 20.7	0.05
**No. of ocytes retrieved per OPU cycle mean±SD**	8.0±6.3	8.1±6.2	8.0±6.7	7.9±6.0	8.0±6.4	NS
**Fertilization rate %**	55.9	55.6	55.9	57.4	54.7	NS
**Number of embryo transfer mean±SD**	188.7 ± 19.6	192.5 ± 15.6	167.2 ± 12.7	190.7 ± 15.2	204.5 ± 17.8	0.03
**No. of embryos transferred per cycle mean±SD**	1.3±1.0	1.4±1.1	1.3±1.0	1.3±1.0	1.3±1.0	NS
**Positive BHCG test per embryo transfer %**	27.6	26.3	29.7	26.8	27.5	NS
**Clinical pregnancies per embryo transfer %**	23.3	21.7	24.3	22.7	24.6	NS
**Miscarriage rate %**	4.3	3.5	6.3	3.4	4.4	NS

Likewise, a separate analysis of the vitrified warmed cycles did not show any significant differences in the monthly or seasonal pregnancy rates (Table 3). Two-way ANOVA comparison of the clinical pregnancy rate in fresh embryo transfer cycles and in vitrified warmed cycles during the consecutive months and seasons did not show any significant statistical difference. Multi variable regression analysis revealed that fertilization treatment parameters such as the number of retrieved eggs and the fertilization rate were significantly influenced by the patient's age (p<0.001) but not by the season distribution (p = 0.43, p = 0.3 respectively).

**Table 3:** Treatment cycles of vitrified-warmed cycles stratified by seasons.

	TOTAL	Spring	Summer	Autumn	Winter	P-VALUE
**Number of warmings**	96.9 ± 21.5	95 ± 30.8	89.2 ± 21.2	102.2 ± 20.2	101.2 ± 18.8	0.84
**Transfers/warmings (%)**	87.7	94.5	88.5	87.7	89.2	0.22
**Clinical pregnancies per transfer (%)**	23	23.1	22.5	23.5	22.4	0.99
**Miscarriage rate %**	5.6	5	6.8	3.4	9.1	NS

## Discussion

Several studies from different parts of the world that examined seasonal fluctuations in IVF treatment outcomes have yielded conflicting results. Moreover, even in studies that found seasonal effect on IVF treatment outcome, the results were not uniform regarding to which season yields optimal pregnancy rates. Stolwijk et al, in their Dutch study, found improved pregnancy rate during the period from November to February [18]. On the other hand, Wood et al studied the IVF and ICSI treatment results in England, demonstrating optimal pregnancy rates in the summer months compared to winter months [9]. In a study of Rojansky et al, better fertilization rate and embryo quality were observed in the winter and spring, although the variance in pregnancy rate did not reach statistical significance [8]. The physiology of the above seasonal variances was explained by the effect of melatonin on the female reproductive tract. In humans, granulosa cells contain receptors to melatonin, which is found in the follicular fluid and is involved in follicular development, ovulation, oocyte maturation, and luteal function. Moreover, it has been suggested that melatonin treatment for patients with infertility, improves oocyte quality [19].

In the present study, we could not demonstrate any significant seasonal influence on the IVF treatment outcome. In the fresh cycles, our results showed no significant seasonal difference in the number of retrieved ovum, fertilization rate or pregnancy rate. Likewise, there was no significant difference in the endometrial receptivity between the seasons, demonstrated by the similar pregnancy rates in the vitrified warmed cycles. This lack of seasonal influence, was also demonstrated in previous studies [11,12,14]. Unlike previous studies, we chose to analyze separately the seasonal effect on fresh embryo transfer cycles and on vitrified warmed cycles. This separate analysis might minimize the complexity of the numerous factors effecting IVF treatment outcome and isolated the effect of endometrial receptivity. The separate analysis also did not demonstrate differences in the treatment outcome during the calendar months nor seasons.

The lack of seasonal influence may be explained by the absolute control of the hypothalamic-pituitary-ovarian function and endometrial receptivity in IVF treatment cycles, making the environmental effects irrelevant. Likewise, seasonal changes in sperm quality are cancelled in IVF due to the use of intracytoplasmic sperm injection procedures.

A limitation of our study is the missing data regarding the patients' characteristics such as number of treatment cycles and etiology of infertility. Likewise, our data does not include the quality grading of the transferred embryos or the day of transfer. Nonetheless, our practice is to transfer day 2–3 top quality embryo and the prevalence of patients that undergo embryo transfer without a top quality grading is less than 10% of transfers. An advantage of our study is the large sample size throughout four years period in a single center and the separate analysis of fresh and vitrified warmed cycles that isolates the endothelial receptivity factor.

In conclusion, our study did not show any significant influence of the calendar months or seasons on the clinical pregnancy rates of fresh or vitrified warmed embryo transfers. It is possible that the complete pharmaceutical control of the ovarian and endometrial function as well as the homogeneous treatment, procedures and laboratory equipment during the study period minimize the influence of seasonal effect on fertilization treatment.

## Supporting information

S1 File Dataset. (XLSX)
